# Cellular Prion Protein and Amyloid-β Oligomers in Alzheimer’s Disease—Are There Connections?

**DOI:** 10.3390/ijms26052097

**Published:** 2025-02-27

**Authors:** Michał Fułek, Naomi Hachiya, Martyna Gachowska, Jan Aleksander Beszłej, Elżbieta Bartoszewska, Donata Kurpas, Tomasz Kurpiński, Hanna Adamska, Rafał Poręba, Szymon Urban, Katarzyna Fułek, Jerzy Leszek

**Affiliations:** 1 Department and Clinic of Diabetology, Hypertension and Internal Diseases, Institute of Internal Diseases, Wroclaw Medical University, 50-556 Wroclaw, Poland; 2 Shonan Research Center, New-STEP Research Center, Central Glass Co., Ltd., Shonan Health Innovation Park 26-1, Muraoka Higashi, Fujisawa 251-8555, Kanagawa, Japan; naomi.hachiya@cgco.co.jp; 3 Faculty of Medicine, Wroclaw Medical University, 50-367 Wroclaw, Poland; martyna.gachowska@student.umw.edu.pl (M.G.); elzbieta.bartoszewska@student.umw.edu.pl (E.B.); tomasz.kurpinski@student.umw.edu.pl (T.K.); 4 Department and Clinic of Psychiatry, Wroclaw Medical University, 50-367 Wroclaw, Poland; jan.beszlej@umw.edu.pl; 5 Division of Research Methodology, Department of Nursing, Faculty of Nursing and Midwifery, Wroclaw Medical University, 51-618 Wroclaw, Poland; donata.kurpas@umw.edu.pl; 6 Department of Rheumatology and Internal Medicine, Marciniak Lower Silesian Specialist Hospital, 54-049 Wroclaw, Poland; hannaklepacz@gmail.com; 7 Department of Biological Principles of Physical Activity, Wroclaw University of Health and Sport Sciences, 51-612 Wroclaw, Poland; rafal.poreba@awf.wroc.pl; 8 Department of Cardiology, The Copper Health Center, 59-301 Lubin, Poland; szymon.urban.wro@gmail.com; 9 Department and Clinic of Otolaryngology, Head and Neck Surgery, Wroclaw Medical University, 50-556 Wroclaw, Poland; katarzyna.fulek@umw.edu.pl

**Keywords:** Alzheimer’s disease, cellular prion protein, amyloid β and PrP interaction in Alzheimer’s, BACE1, Aβ

## Abstract

Alzheimer’s disease (AD) is the most common cause of dementia worldwide. Pathological deposits of neurotoxin proteins within the brain, such as amyloid-β and hyperphosphorylated tau tangles, are prominent features in AD. The prion protein (PrP) is involved in neurodegeneration via its conversion from the normal cellular form (PrPC) to the infection prion protein scrapie (PrPSc) form. Some studies indicated that post-translationally modified PrPC isoforms play a fundamental role in AD pathological progression. Several studies have shown that the interaction of Aβ oligomers (Aβos) with the N-terminal residues of the PrPC protein region appears critical for neuronal toxicity. PrPC-Aβ binding always occurs in AD brains and is never detected in non-demented controls, and the binding of Aβ aggregates to PrPC is restricted to the N-terminus of PrPC. In this study, we aimed to gather all of the recent information about the connections between PrPC and AD, with potential clinical implications.

## 1. Introduction

Pathological aggregates of the amyloid-β (Aβ) peptide and hyperphosphorylated tau are the main changes responsible for developing AD, so the question of possible interactions between them seemed obvious. Indeed, a lot of studies confirm the relationships between those molecules [1,2,3,4]. Not only do monomeric and oligomeric Aβ interact with phosphorylated tau in AD neurons, but what may be even more important is that these interactions progressively increase with the progression of AD [5]. These interactions may cause structural and functional damage, particularly if the interaction occurs at synapses [6]. Because of the ability to bind directly to the receptor, soluble Aβ and tau impair synaptic plasticity, leading to neurite degeneration and the activation of kinases, including Fyn, which itself can enhance tau phosphorylation [1].

The major components of Aβ aggregates in the AD brain are neuritic plaques, diffuse amyloid, and vascular amyloid. The aggregation of Aβ in the form of amyloid fibrils has long been considered central to the pathogenesis of AD [7,8].

However, in recent years, a large body of evidence has strongly supported that soluble Aβos are more detrimental to synaptic plasticity than the Aβ that causes amyloid fibril formation. Soluble Aβos appear to be more potent than fibrillar Aβ aggregates for the transmission of Aβ pathology [9]. Their ability to inhibit long-term potentiation (LTP) and many other critical neuronal activities is responsible for the classic model of synaptic plasticity and memory loss in vivo, for example, in middle-aged Tg2576 mice, which are equipped with a variant of human amyloid-β precursor protein (APP) associated with AD and in culture cells [9,10,11,12]. These studies stoutly defend the idea that soluble Aβos are the causative agents of AD.

Furthermore, soluble Aβos have been found to be very high in the AD brain, and their levels correlate strongly with the severity of the disease [13]. The results of clinical trials on mild AD subjects with solanezumab, the antibody that binds to soluble monomers (not to plaques), suggested a statistically significant cognitive benefit [14]. Ample evidence is indicative of the direct attribution of decreased hippocampal LTP and altered memory function to an isolated, biochemically defined, assembly form of human Aβ soluble oligomers in the absence of amyloid fibrils [15]. By binding to receptors on the surface of neurons, Aβos are thought to initiate signaling pathways that lead to synaptic dysfunction and neuronal death. Interestingly, the mechanism by which Aβos exert their toxic effects is related to PrP, the etiologic protein of prion diseases [16,17] and a glycoprotein in cell membranes [8,18]. As shown in Table 1, the cellular prion protein (PrPC) serves as a potential receptor for Aβos. PrP is ubiquitously expressed but concentrated in the central nervous system of the brain and spinal cord, where it functions as a receptor that can mediate the neurotoxic effects of Aβos [19,20,21].

The aim of this paper is to show the latest findings of the clinical pathology of AD changes depending on the interaction between PrP and Aβ [8,22,23].

**Table 1:** Comparison of PrPC’s and Aβ’s characteristics.

Characteristics	Cellular Prion Protein	Amyloid-β	References
**Structure**	N-glycosylated anchored protein GPI	Short peptide derived from amyloid precursor protein	[24] [25]
**Localization**	The surface of the neuron cell	Extracellular matrix	[24] [25]
**Role in pathogenesis**	Potential receptor for Aβos, facilitating neuronal toxicity	Aggregates into insoluble fibrils, exacerbating neurotoxicity and neurite damage	[25] [7]
**Modifications according to the progression of the disease**	Increased proportion of unglycosylated PrPC in AD relative to MCI or NCI	Dysregulated Aβ metabolism, with production exceeding clearance, promotes fibril formation and neurotoxic accumulation	[26] [27]

## 2. Alzheimer’s Disease

AD is a multifactorial illness that causes cell degeneration in the brain and is the leading cause of dementia, which is defined by a loss of cognitive ability and independence in everyday tasks [28]. Nowadays, over 55 million people struggle with dementia, and 60–70% of them suffer from AD. It is estimated that every year, there are approximately 10 million new cases of dementia worldwide. This disease is currently assessed to be the seventh major cause of mortality and one of the primary reasons for disability and reliance among the elderly [29]. Moreover, the prevalence of dementia is expected to double in Europe and triple globally by 2050, according to the most recent data. This projection is tripled when considering a biological definition of AD instead of a clinical one [30].

Symptoms of AD vary and can be very individual, though the most prevalent first signs involve memory problems. Some people experience mild cognitive impairment (MCI), which means greater memory issues than typical for their age. It may also be associated with issues related to movement and smell. Alzheimer’s disease is more likely to strike older adults with MCI [31]. Early symptoms include memory impairment (difficulties with short-term memory), cognitive decline (challenges in word-finding, spatial navigation, and reasoning), and neuropsychiatric symptoms (depression, apathy, and subtle mood changes). Intermediate symptoms include progressive cognitive dysfunction, language and communication issues, behavioral changes, and social and functional decline. In the final stages, patients display severe cognitive decline, loss of motor function, progression of behavioral changes, and dependence on caregivers to meet basic needs [32,33].

Patients can also exhibit clinical features specific to atypical phenotypes of AD, such as visual–spatial deficit in posterior cortical atrophy AD, language disability in logopenic variant primary progressive aphasia, behavior-predominant or dysexecutive syndromes in behavioral AD and dysexecutive AD, and motor dysfunction in corticobasal syndrome AD [34].

## 3. Cellular Prion Protein and Alzheimer’s Disease

### 3.1. Structure and Function of PrPC

Cellular prion protein is a small protein encoded in the *PRNP* gene on human chromosome 20. It is a glycosylphosphatidylinositol (GPI)-anchored protein that can be found in several types of cells, most prevalently in the central nervous system (CNS). It plays various roles in cellular function [35]. It has become known due to being a crucial part of the pathogenesis of prion diseases, which are neurogenerative disorders [36]. PrPC serves as both a substrate for prion production and a modulator of prion toxicity throughout the course of all prion disorders [37]. It displays a high affinity toward the binding side for Aβos on the neuronal membrane [38]. Moreover, it promotes carcinogenesis by tumor growth regulation, differentiation, and resilience toward conventional treatment [39].

PrPC has a modular structure comprising an intrinsically disordered N-terminal domain and a structured C-terminal globular domain. The first one (amino acids 23–121) is flexible and has the ability to bind metal ions, such as copper [40]. The other region (amino acids 127–231) is built of three α-helices and two β-strands and is responsible for GPI anchoring. After *PRNP* gene transcription, the peptide is created via endoplasmic reticulum-attached ribosomes and the GPI anchor is added. The final form of PrPC has 208 residues and 5 octapeptide repeats at its N-terminal domain and hydrophobic domain in the middle. This protein may undergo glycosylation at two strongly conserved amino acid locations (N180 and N196) to produce diglycosylated, monoglycosylated at N180 or N196, or unglycosylated forms [35]. PrPC may be driven to refold into a different conformation, creating PrPSc, which is internalized via a PrPC-mediated process, causing transmissible spongiform encephalopathies, known as prion diseases [41].

### 3.2. Neuronal Function of PrPC

PrPC is located mainly on the surface of neurons, anchored by a GPI moiety. It is especially abundant in synaptic regions, contributing to processes like synaptic plasticity and neuroprotection. This protein can also be found in astrocytes and oligodendrocytes, where it takes part in glial cell signaling and support. PrPC is distributed throughout the brain, with higher concentrations in regions such as the hippocampus, cerebellum, and cerebral cortex [42]. It is thought to have a variety of roles in the nervous system, including protection against ischemia shock, apoptotic agents, and reactive oxygen species (ROS). Moreover, it is also involved in neuronal transmission preservation, synaptic plasticity, neurite outgrowth, circadian rhythm, maintenance of peripheral myelin during aging, memory, and motor behavior [43]. Understanding the structural features of PrPC is essential to elucidate its interaction with Aβos and its contribution to neurotoxicity in AD.

### 3.3. Interaction of PrPC with Other Proteins

PrPC functions as a cell surface receptor for Aβ, as previously mentioned, as well as for other neurodegeneration-associated proteins, including α-synuclein, superoxide dismutase 1 (SOD1), and TAR DNA-binding protein 43 (TDP-43). PrPC mediates the neurotoxic effects of α-synuclein by promoting Fyn kinase phosphorylation, leading to neurite damage and dysfunction. Additionally, PrPC facilitates the internalization of α-synuclein, potentially influencing its aggregation and propagation within neuronal cells [44]. PrPC also plays a regulatory role in SOD1 activity, contributing to the reduction in oxidative stress in brain tissue, thereby manifesting antioxidant properties. Notably, research has demonstrated that in the early stages of AD, PrPC levels are highest in brain regions experiencing the greatest oxidative stress burden [45]. Similar to its interaction with α-synuclein, the interaction between PrPC and TDP-43 promotes neurite damage by enhancing protein uptake. Studies indicate that elevated PrPC expression on the cell surface correlates with increased TDP-43 internalization and the formation of intracellular fibrillar aggregates [44,46].

### 3.4. PrPC in Alzheimer’s Disease Pathology

**Figure 1:**
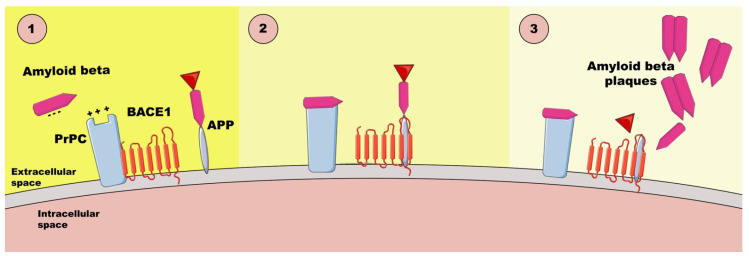
The interactions between Aβos and PrPC based on [47]. PrPC—cellular prion protein, BACE1—β-amyloid precursor-cleaving enzyme 1, APP—amyloid-β precursor protein.

PrPC can also act as a receptor or receptor or co-receptor for Aβos [47], which is important in AD pathology (Figure 1). It binds Aβos through specific domains, mediating downstream toxic effects such as synaptic dysfunction and neuronal damage [38]. The binding of negatively charged Aβos and the positively charged N-terminal of PrPC causes the hyperphosphorylation of tau, resulting in synaptic destruction. The damage caused by the binding of Aβos to synaptic prions, which reside on membrane lipid-rich rafts, leads to synapse disintegration, cognitive failure, and cell death [47]. This interaction involves signaling pathways, such as Fyn kinase activation, contributing to impaired synaptic plasticity and memory deficits [38]. Moreover, RNA aptamer, which is negatively charged, can promote the release of Aβ from the Aβos-PrPC complex by engaging two positively charged patches at the PrPC′s N-terminus [47]. Understanding PrPC’s role in Aβo binding offers insights into therapeutic targets for mitigating Alzheimer’s disease progression [38].

There are some conflicting results regarding the elevation of membrane-binding PrPC levels in the brain tissue of AD patients compared with patients with MCI or no cognitive impairment (NCI). The discrepancy may arise due to the lack of specificity of the assay for the prion’s protein isoform [26].

One study found that the soluble Aβ assemblies derived from the brains of individuals with AD interacted with PrPC at the postsynaptic density to activate the Src kinase Fyn, which phosphorylates the NR2B subunit of NMDA receptor and causes a transient increase in NR2B on the cell surface with consequent excitotoxicity while rendering the destabilization of dendritic spines. This molecular mechanism of PrPC-mediated Aβ toxicity indicates a prion connection between Aβ and Fyn [48]. Another study demonstrated that soluble Aβ binds to PrPC at neuronal dendritic spines, where it forms a complex with Fyn and results in the activation of the kinase and subsequent Fyn-dependent tau hyperphosphorylation in a PRNP gene dose-dependent manner, making another prion connection [49].

The metabotropic glutamate receptor, mGluR5, a transmembrane protein in the postsynaptic density, could be another protein involved in Aβo-PrPC binding, linking Aβo-PrPC to Fyn [28]. Some data point out that agents, like caveolin-1 or the neural cell adhesion molecule (NCAM), could potentially connect PrPC and Fyn from the two opposite sides of the cell membrane [50,51,52].

1. Electrostatic attraction—negatively charged Aβos in the interstitial fluid are attracted to positively charged patches on PrPC.
2. Selective binding—Aβos bind to PrPC via a cationic patch produced by the folding together of the 23–27 and 92–111 regions. Disruption of PrPC-BACE1 complex—PrPC normally interacts with BACE1 (β-amyloid precursor-cleaving enzyme 1) to inhibit Aβ42 production; Aβo binding disassociates PrPC from BACE1, leading to the activation of the enzyme and increased production of Aβ42, thereby activating the APP-cleaving enzyme.
3. Cascade of synaptic damage—Aβ x–42 → Aβos → toxic hyperphosphorylated tau oligomers, which spread through the entorhinal cortex to the neocortex and the subcortical control panels.

## 4. PrPC and AD Stages

PrPC isoforms become modified in various pathological processes of AD. The diverse phenotypes of PrPC appear to be risk factors for either the slow or rapid progression of the disease. The specific PrPC isoforms participate in the association between modified PrPC interacting proteins and AD pathology. The association between the glycosylation pattern of PrPC and the severity of AD may eventually be a potential diagnostic biomarker for the pathology. A growing body of literature has indicated that PrPC deposits often accompany Aβ plaques in AD and that PrPC was the high-affinity receptor to Aβ42 oligomers on cells. The altered expression of PrPC seems to be associated with disease progression. The finding that PrPC is decreased in the hippocampus and temporal cortex in aging and sporadic AD but not in familial AD supports the hypothesis that reduced PrPC expression reflects a main mechanism of disease and is not a consequence of other AD-associated changes [53].

Other studies have shown changes in PrPC expression levels in the late stages of AD, probably due to the loss of neurons. PrPC protein expression in the brain increases in the initial stages of AD and reaches its peak around stage III. Henceforward, PrPC expression declines up to the clinical manifestation of the disease [54]. The impingement of Aβos with the N-terminal residues of the PrPC protein region appears critical for neuronal toxicity. PrPC-Aβ binding is regularly present in AD brains, but it has not been found in non-demented controls. The N-terminal residues 23–27 and the 92–110 region seem to be critically important for PrPC interactions with Aβ42 oligomers because the deletion of either of these regions results in a significant loss of binding. Some preliminary work in this field also suggests that N-terminal residues 23–27 and the 95–110 region of PrPC incorporate the strategic amino acid binding sequence for oligomer Aβ-induced synaptic deterioration and apoptosis of the neurons. The mentioned fragments of PrPC strongly impede the Aβ42’s both cyto- and synaptotoxic efficiency [55].

## 5. PrPC and AD Prevention

Interactions with the APP-cleaving enzyme BACE1 through its N-terminal polybasic domain is another function of PrPC that inhibits enzyme activity, resulting in a reduction in Aβ production, which indicates a preventive role against AD [56]. Membrane-binding PrPC has been demonstrated to regulate LTP in the hippocampus, which is induced by oligomeric Aβ42. Recently, it was found that PrPC can interact with the different forms of Aβ, like synthetic Aβos—Aβ-derived diffusible ligands (ADDLs). Additionally, PrPC has also been shown to bind to ADDLs, which are tightly related to cognitive impairment in multiple mouse models of AD. PrPC is a major component for the inhibition of LTP by ADDLs from AD brains [57,58].

The PrPC-Aβos interaction is dependent on raft-based complexes. The most important for the interaction between Aβ42 and PrPC is cholesterol-rich lipid rafts. GPI-anchored PrPC is localized to the cholesterol-rich lipid raft microdomains of the plasma membrane. Cholesterol depletion disrupts these rafts, with PrPC being redistributed into the non-raft regions of the membrane. The disruption of the rafts causes a significant reduction in Aβo binding to cells and prevents the activation of Fyn kinase. Studies indicate that PrPC functions as an extracellular scaffolding protein that is able to organize multiprotein complexes that mediate intracellular signal transduction at the cell surface [59].

## 6. Interactions

### 6.1. Aβ Transmission

The accumulation of misfolded Aβ in the brain is a significant neuropathological characteristic of AD. It has recently been demonstrated that this Aβ pathogenic alteration, like prions, can be passed between individuals. Inter-individually transferred Aβ has also been found to be deposited in cerebral blood vessels. Kane et al. injected AD patient brain homogenate into the brains of 3-month-old APP transgenic mice in 2000 (Tg2576) [60]. At 5 months (8 months), they discovered much higher Aβ deposition in the brain parenchyma and cerebral blood vessels than in the control group. This is the first report of the possibility of transmitting cerebral Aβ amyloidosis from one person to another [61]. This result was then repeated in various AD model mice (APP23, APPPS1) as an inter-individual transmission investigation employing animals with transgenic mutations [62].

Furthermore, the fact that Aβ propagation is dependent on the time since Aβ injection and the concentration of Aβ injected, as well as the fact that Aβ propagation does not occur when Aβ-removed brain homogenates are employed, demonstrated that Aβ is required for this propagation [62]. Furthermore, Aβ fibers made from synthetic Aβ and injected into the brains of genetically altered mouse models have been shown to generate Aβ deposition in the brain [63,64]. In these Aβ propagation experiments, the neuropathological phenotype and biochemical properties of deposited Aβ are determined by the host AD model mouse and the type of brain homogenate injected. This closely resembles the phenotype of prion diseases, which is determined by variations in PrPSc strains [62,65,66].

### 6.2. Propagation

The morphologies of Aβ fibers produced in vitro under various circumstances vary. Pathological brain amyloidosis was described differently for each Aβ fiber shape when distinct Aβ fibers were introduced into the brains of AD model mice [63]. Furthermore, when homogenates of patient autopsy brains with different APP gene mutations were injected into the brains of AD model mice, they demonstrated varied clinical characteristics [66]. According to these reports, Aβ, like PrPSc, has strains. Furthermore, it was demonstrated that both the insoluble portion of Aβ fibers that are not destroyed by proteinase K (PK) and the soluble fraction of Aβ that PK degrades can promote propagation. This implies that more than one form of Aβ is responsible for propagation. Sonication breaks down the insoluble part of Aβ into smaller soluble fractions, increasing propagation efficiency [61,67].

Experiments on the peripheral spread of Aβ cerebral amyloidosis indicated that Aβ cerebral amyloidosis is transmitted by intraperitoneal administration of brain homogenate, showing that Aβ cerebral amyloidosis, like prion, can be spread by peripheral administration. According to these observations, Aβ transferred from the periphery was first deposited in the vessel wall before spreading to the brain parenchyma [68,69]. Inter-individual transmission was found to occur in mice transgenic with the human APP gene that does not have a genetic mutation. Although these mice did not exhibit spontaneous Aβ deposition in the brain, intracerebroventricular inoculation of brain homogenates from AD patients did result in Aβ deposition in the brain 285 days after inoculation. Furthermore, Thioflavin S-positive Aβ deposition was not found until 450 days following inoculation, whereas Aβ deposition was observed at 585 days [70]. The study showed that, like prion illnesses, genetic changes are not required for inter-individual transmission of cerebral amyloidosis.

### 6.3. Medical Practice

By 1985, when the risk of prion infection from postmortem brain-derived hGH injections was first recognized, about 30,000 children worldwide with growth abnormalities, such as short stature, had received intramuscular hGH injections. By 2012, there had been 226 incidences of lethal illness. France had the most outbreaks (119 cases), followed by the United Kingdom (65 instances) and the United States (29 cases). Four of eight young patients (36–51 years old) with growth hormone-associated Creutzfeldt–Jakob disease (CJD) demonstrated moderate to severe Aβ deposition in the brain parenchyma and cerebral arteries in studies conducted using autopsy brains. The growth hormone preparation-related CJD group had considerably more advanced Aβ deposits than the other 19 prion disease cases (aged 36–51 years). Before death, none of the individuals displayed clinical signs of AD. Furthermore, amyloid lesions in the brain were uncommon at such a young age, and none of the eight patients who died had genes predisposing them to early-onset AD or other neurodegenerative disorders. As a result, it is likely that the hGH injections introduced aggregated nuclei of Aβ amyloid, similar to prions in CJD. Based on these findings, the most logical explanation is that the amyloid lesions detected in the brains of CJD patients who had previously received hGH therapy were propagated and generated by hGH extract preparations contaminated with aggregating amyloid-β nuclei [71].

The most common form of iatrogenic CJD is produced by the transplantation of prion-contaminated cadaveric dura mater (dried human cerebral dura mater) into a dural defect site following neurosurgery. Post-dural transplant CJD cases had more advanced cerebral amyloid angiopathy (CAA) in the meninges and Aβ deposition in the submucosa than isolated CJD cases, according to a study using autopsy brains, and the severity of these symptoms was positively correlated with the time from dural transplant to death. As a result, it is hypothesized that in dural transplantation, Aβ deposition spreads from the brain’s surface due to the direct placement of the Aβ-contaminated transplanted dura mater on the brain’s surface [72]. Furthermore, the immunostaining of the dura of 84 elderly patients (median age 84.9 years) revealed Aβ deposits in 13% of cases [73]. As a result, these data suggest that medical therapy may have spread cerebral amyloidosis.

Prions are more difficult to eliminate than bacteria or viruses. Prions adhere to metals tightly and necessitate strict sterilization procedures to remove contamination, which might harm medical tools. If AD is proven to be transmitted via the same mechanism as prions, the impact on public health and surgical practice will be huge, and a considerable price will have to be paid. Recent articles, on the other hand, have reported on the effectiveness of autoclaving against Aβ [74]. Both prion and Aβ decontamination procedures must be thoroughly and quickly investigated, as both can be passed from person to person.

## 7. PrPC in AD Therapy

Several therapeutic approaches targeting PrPC in AD have already been proposed, due to their role in binding to Aβos and mediating downstream neurotoxic signals. In Table 2, we summarize the recent approaches, which include monoclonal antibodies directed at PrPC, fragments of PrPC, small molecules, gene therapy, and indirect approaches. The strategies aim to mitigate the toxic effects of Aβos while addressing the complexity of AD pathology. While each approach offers unique advantages, challenges, such as delivery to the brain, toxicity, and efficacy, remain to be overcome.

### 7.1. Monoclonal Antibodies

Monoclonal antibodies, such as 6D11 and 8G8, have been proven to inhibit the binding of Aβos to PrPC. These antibodies specifically target the amino acid segment 95–105 in PrPC, a critical region for Aβo recognition. The 6D11 antibody demonstrated efficacy in preventing Aβo-induced LTP suppression in the hippocampus of wild-type mice. Additionally, it inhibits abnormal phosphorylation of Fyn kinase, the NR2B glutamate receptor subunit, and tau protein [75].

In a study by E. Chung et al., the intraperitoneal administration of 6D11 in aged APP/PS1 mutant transgenic mice not only rescued memory deficits but also increased hippocampal synaptophysin levels. However, further research is needed to establish the in vivo efficacy of anti-PrPC antibodies without toxicity. Notably, the efficient elimination of behavioral deficits in mice requires a high dose of 400 mg/kg over 12 days, and the pharmacokinetics and utility of human anti-PrPC antibodies for clinical dosing remain undefined [76].

While neuronal toxicity has been reported in some cases, a study by T. O. Cox et al. showed that it can be mitigated by adding a second antibody targeting the natively unfolded N-terminus of PrPC, overlapping with the AZ59 epitope [77]. Their research revealed that the systemic administration of a murine version of a human anti-PrPC N-terminal antibody at clinically relevant doses did not result in toxicity. Safety was confirmed through behavioral testing, hippocampal synaptic density analysis, and assessments of astrocytosis and microgliosis. Interestingly, despite memory recovery, this approach did not significantly alter dense-core plaques or soluble Aβ oligomers, suggesting that combination therapies targeting Aβ accumulation and gliosis may offer the greatest benefits [77].

Other antibodies, such as ICSM-35 and ICSM-18, which target the 95–105 region and α-helix 1 of PrPC, have also been shown to block Aβ oligomer–PrPC interactions and prevent Aβ oligomer-induced LTP suppression [78]. These findings reinforce the potential of monoclonal antibodies as therapeutic agents, though challenges related to dosing, toxicity, and translation to clinical settings remain [76,78].

### 7.2. Fragments of PrPC

A novel therapeutic approach focuses on targeting soluble fragments of PrPC, particularly the N-terminal fragment (N1), which has the ability to bind Aβos. Acting as a decoy receptor, N1 can neutralize Aβos and reduce their neurotoxic effects. Studies have shown that N1 protects primary neurons from toxicity induced by both engineered and AD patient-derived Aβos. Moreover, in mice exposed to Aβos, N1 has been observed to prevent synaptic damage in hippocampal neurons and protect against memory dysfunction [79]. Notably, N1 has been demonstrated to suppress Aβo-induced cytotoxicity in primary neurons and prevent the inhibition of LTP in the hippocampus. Beyond its direct effects on Aβos, soluble PrPC fragments modulate Aβ fibrillation, potentially reducing its toxicity. This opens pathways for the development of biomarkers to monitor therapeutic target modulation in vivo [80,81].

### 7.3. Small Molecules

Another approach includes the usage of small molecules and peptides targeting PrPC and preventing the Aβos-PrPC interaction. The potential benefit is associated with the passage of small molecules through the blood–brain barrier (BBB) and easier access to the nervous system. Interestingly, not many in vitro-studied small molecules targeting the PrPC were proven to be effective in in vivo studies. Antiprion activity was shown in many in vitro studies via prion inhibitory assays in cell culture using sulfated polysaccharides, amphotericin B, anthracycline, phthalocyanines, porphyrins, pentosan polysulfate, quinacrine, and memantine [82]. Quinacrine and pentosan polysulphate, for which great hopes were attached, have been withdrawn as infective in patients [83,84].

### 7.4. Gene Therapy

In prion disease therapy, strategies inhibiting PrPC expression have shown promise in interfering with prion propagation [85]. In transgenic mice in which the PRNP gene was knocked out during the early stage of prion disease, a reversal of early spongiform changes was observed. Moreover, blocking the expression of the PRNP gene using lentivector-mediated post-translational gene silencing mediated by RNA interference was shown to reduce neuronal PrPC expression, shedding new light on its potential use also in AD therapy [86]. Importantly, PrPC also plays a role in protection against oxidative stress; therefore, the complete invalidation of PrPC could also bring negative consequences, such as increased susceptibility to oxidative damage [87].

### 7.5. Indirect Pathways

Currently, Mastinib is used in phase III studies for the treatment of AD, which is a phenylaminothiazole-type tyrosine kinase inhibitor. Although it does not directly target PrPC, its mechanism of action includes the inhibition of Fyn kinase. PrPC interacts with Fyn-mGluR5, forming a postsynaptic signaling unit. When Aβ is bound to this complex, it leads to Fyn activation. This interaction causes the phosphorylation of the N-methyl-D-aspartate receptor (NMDAR) and leads to the loss of NMDARs from the neuronal surface. This results in the dysregulation of synaptic transmission and neuronal impulse propagation, contributing to cognitive impairment. Mastinib, as a Fyn kinase inhibitor, prevents PrPC-Fyn activation [88,89].

Noteworthily, in 2024, the European Medicines Agency (EMA) approved lecanemab, a monoclonal antibody for treating MCI and early-stage AD. The drug is indicated for patients with Aβ plaques in the brain and one or no copies of the ApoE4 allele [90]. Lecanemab binds to soluble Aβ protofibrils, the most neurotoxic form of Aβ. Phase 3 trials demonstrated its efficacy in reducing amyloid biomarkers and moderately slowing cognitive and functional decline. Unlike previous AD therapies, which primarily addressed symptoms, lecanemab targets the underlying pathophysiology, representing a potential breakthrough in altering disease progression [91].

While there is currently no direct evidence or established connection between lecenemab and PrPC in the literature, studies should investigate how the PrPC-Aβ interaction is influenced by this monoclonal antibody. The therapeutic strategies including both lecanemab and PrPC could potentially complement each other. PrPC, acting as an Aβ-receptor, mediates neurotoxicity, including synaptic dysfunction and cognitive impairment. Since lecanemab targets soluble Aβ protofibrils, it may reduce the pool of Aβ oligomers available to interact with PrPC, potentially leading to a reduction in the neurotoxic effect. Moreover, targeting different aspects of Aβ toxicity could enhance therapeutic efficacy [91,92].

### 7.6. Limitations

Exploring potential molecular therapies in AD requires an understanding of the existing limitations and side effects. Monoclonal antibodies, while effective in preclinical studies, are associated with high toxicity due to high doses. Gene therapy must balance PrPC silencing with preserving its protective role. Additionally, the BBB is a major obstacle to delivering therapeutic molecules. Due to the complexity and multifactorial nature of AD, it remains a challenge to intricate all the interactions, overlap pathways, and individualize the variability in disease progression. The balance between the risks and benefits should be carefully considered. For creating personalized treatments and improving outcomes, the collaboration between scientists and clinicians is crucial [76,93,94].

**Table 2:** Potential pharmacological strategies for AD treatment.

Treatment	Mechanism	References
**Monoclonal Antibodies**	Inhibition of the binding of Aβ and PrPC; Prevention of Aβo-induced LTP; Inhibition of Fyn kinase phosphorylation.	[76]
**Fragments of PrPC**	Neutralization of Aβos; Modulation of Aβ aggregation dynamics, reducing fibril formation; Reduction in Aβ-mediated neurotoxicity.	[95]
**Small molecules**	Inhibition of the interaction between Aβos and PrPC.	[82]
**Gene therapy**	Suppression of PrPC expression via targeted silencing of the *PRNP* gene.	[85]
**Indirect pathways**	Direct inhibition Fyn kinase activity; Prevention of PrPC-Fyn kinase activation.	[89]

### 7.7. PrPC as a Biomarker—Diagnostic Possibilities

Investigating the concentration of PrPC in cerebrospinal fluid among patients with neurodegenerative disorders, including AD, has shown to be a potential diagnostic tool for neurodegenerative disorders. Studies have demonstrated that PrPC levels are reduced in patients with neurodegenerative diseases compared to healthy individuals. Moreover, a significant correlation was observed between the PrPC concentration and the severity of cognitive decline, suggesting its utility in monitoring disease progression [96,97].

However, other studies report no definitive association between PrPC levels in CSF and the cognitive status of AD patients. These conflicting findings indicate that while the hypothesis of PrPC serving as a biomarker is promising, it remains inconclusive. To validate the hypothesis of whether PrPC concentration in CSF can reliably predict AD diagnosis or progression, research must still be performed [98].

## 8. Conclusions

Interactions between PrPC and Aβos are one of the important molecular mechanisms that play a role in the pathogenesis of AD. Studies suggest that PrPC acts as a receptor for Aβos, mediating the neurotoxic effects including synaptic dysfunction, dendritic spine loss, and cognitive impairment. Additionally, shifts in the glycosylation profile of PrPC have been proven as a pathological agent and a potential biomarker in AD progression. Therapeutic strategies targeting PrPC (monoclonal antibodies, PrPC fragments, small molecules, gene therapy) offer promising tools to mitigate the neurotoxic effects of Aβos. However significant challenges, such as delivery across BBB, toxicity, and maintaining the protective role of PrPC, remain, emphasizing the need for further research. Understanding the variability in PrPC isoforms and their impact on disease progression remains an important area for further exploration.

While the connection between PrPC and Aβ is intriguing, many unknowns remain. PrPC’s ability to influence Aβ toxicity on a molecular level is still not fully understood and needs to be investigated further. Additionally, more studies are required in order to learn more about PrPC’s interactions with other disease factors, such as tau proteins and neuroinflammation. Moreover, the variation in research methods makes it difficult to draw valid conclusions, so standardization is needed. Long-term studies on animal models and clinical trials could play a significant role in developing new AD therapeutical strategies. Unfortunately, there is a risk of overestimating the role of PrPC in AD by scientists, making them overlook other key aspects. Furthermore, there is also a need to determine whether PrPC-targeted treatment could be beneficial for patients suffering from AD, as the therapy side effects could disrupt PrPC’s essential role in brain function.
